# Social integration, physical and mental health and subjective well-being in the floating population—a moderated mediation analysis

**DOI:** 10.3389/fpubh.2023.1167537

**Published:** 2023-07-06

**Authors:** Chengcheng Fei, Yiying Zhu, Longyuan Jiang, Haixia Zhou, Haiyan Yu

**Affiliations:** School of Public Health and Management, Wenzhou Medical University, Wenzhou, China

**Keywords:** social integration, subjective well being, floating population, mental health, social capital, physical health

## Abstract

**Background:**

Individuals of domestic migrant populations in China (specifically, migration that is economically driven) often face difficulties in social integration. They are suffering from discrimination and unfair treatment in work and life, which do harm to their physical/mental health and Subjective Well-Being (SWB).

**Methods:**

The current study utilized a stratified sampling survey in the Yangtze River Delta region of China, in October and November 2022. Six hundred and eleven useful self-reported questionnaires were collected. Questionnaires include questions about social integration, social capital, physical/mental health, and SWB; Bootstrapping method was used to test the mediating effect of physical health and mental health. Multiple hierarchical regression was used to test the moderating effect of social capital.

**Results:**

Social integration had positive impact on the SWB (*r* = 0.523, *p* < 0.01). Bootstrap analysis showed that physical health and mental health partially mediated the correlation between social integration and SWB of Floating Population with a mediation effect of 0.149 and 0.192. Social capital can positively moderate the relationship between two pair of variables: social integration and SWB (*β* = 0.152, *t* = 4.42, *p* < 0.001), physical health and SWB (*β* = 0.148, *t* = 4.39, *p* < 0.01). However, social capital does not play a significant moderating role in the association between the effect of mental health on SWB (*β* = 0.032, *t* = 0.973, *p* > 0.05).

**Conclusion:**

This study proved a significant correlation between social integration and SWB of Floating Population, with physical/mental health playing a mediating role. Enhancing the social integration of floating population and keeping healthy physically and mentally are key to improving their SWB.

## Introduction

*The World Migration Report 2022* states that most of immigrants are socially disadvantaged, often facing higher health risks and lower well-being. Subjective well-being, a dimension of well-being, is an overall evaluation of emotional and cognitive quality of life, including emotional balance and life satisfaction (1). Studies on the SWB of immigrants falls into two main categories. The first category mainly focuses on the basic situation of immigrants’ SWB and the differences between different population types (2–4). For instance, due to objectively poorer living conditions and lower working incomes, immigrants tend to have a weaker sense of life satisfaction and well-being than that of local residents (5). The second category is mainly to study the factors that influence the SWB of immigrants (6–8). The three common factors that affect immigrants’ SWB are personal characteristics, social integration and health (9, 10). Social integration is an important determinant of SWB in this population, because the primary challenge faced after migration is how to integrate into the local society as quickly as possible. As these individuals begin to integrate into the local society in terms of education, work, and life, problems such as discrimination, unequal treatment, and lack of social security inevitably arise, which directly and/or indirectly affects life satisfaction, and thus, SWB (11, 12). In addition to social integration, physical and mental health are also important factors affecting the SWB of immigrants (4, 10). This is because immigrants do not have equal access to healthcare services and are exposed to higher work stress after migration, which leads to a decline in health status, worsening their emotional experience, and ultimately leading to lower well-being (13). At the same time, the stress caused by differences in cultural practices can affect the mental health, leading to adverse consequences, such as anxiety and depression, which significantly reduces their emotional evaluation of the quality of their life, thus reducing SWB (14). In fact, research on the SWB of the Floating Population is analogous to that of international immigrants.

Floating Population is a phrase developed under the household registration system (a system to define where people come from) in the past 40 years in China. It refers to domestic population who have left their domicile and migrated from countryside to prosperous areas in search of work. With the advancement of economy and urbanization, floating population has become a nonnegligible group. According to the seventh national population census in 2020 in China, the amounts of Floating Population has increased to 380 million. It is reported that Floating Population often face difficulties in social integration though it’s an important population in China (15). Some factors like personal characteristics, income and level of education can affect floating population’s social position. Normally, floating population are limited in many aspects, such as lack of local welfare, resources and supports (16). They are suffering from discrimination and unfair treatment in work and life, which is detrimental to their health and Subjective well-being.

Given the above information, this article aimed to find how social integration directly or indirectly affects the SWB of the floating population in China. The first feature of this study is that it focuses specifically on China’s domestic floating population, rather than on international immigrants. The second feature is that this study constructs a mediation model that focuses on exploring the mediating effect of physical and mental health between social integration and SWB of floating populations. The third feature of this study is that it explores whether social capital is the moderating factor in each path of the mediation model. Finally, this study will also propose corresponding solution strategies based on the findings to alleviate adverse health consequences, so as to improve the SWB.

## Literature review and hypotheses

Social integration is considered as an important determinant of the floating population SWB (4, 17). Some scholars have studied the degree of social integration in five dimensions: physiological adaptation, economic integration, social adaptation, identity, and psychological integration (18). Most of previous studies on the relation between social integration and SWB have shown a positive relationship between them; that is, individuals with better social adaptation show higher levels of well-being (17, 19).

Physical health is defined as objectively good physical condition and subjectively perceived health (20). To address this factor, economic adaptation, one dimension of social integration, promotes the social class and increases Floating Populations’ utilization of health services, thus protecting their basic health rights and improving their physical quality (21). In addition to the above-mentioned effects of social integration on physical health, physical health also has significant effects on SWB in the following two aspects. When physical health is impaired, the pain and suffering caused by illness often prevents individuals from pursuing happiness, which in turn causes their life satisfaction to plummet (22).

Mental health can be influenced by both physiological and cultural adaptation (23). First, physiological adaptation can help the floating population overcome the problems of acclimatization, help relieve depression, anxiety, and other symptoms, and promote the improvement of mental health (24). Second, acculturation can weaken the psychological stress caused by cultural differences and enhance the positive emotional experience of the floating population, thus increasing their sense of well-being (23, 25). In addition to the above-mentioned effects, mental health also has significant effects on SWB in the following aspect. The employment pressure and economic stress faced by the floating population can cause problems, such as frustration and depression, which eventually lead to adverse emotional experiences (26).

Social capital includes social networks, reciprocity norms, and social trust, which are the social resources brought about by people’s position in the social structure (27). The floating population needs to continuously develop their own social network in the process of social integration (28). In this sense, the floating population is a particularly vulnerable group, because their social network stays in the place of outflow, and it can take years to establish a reliable social network in the place of inflow (29). In terms of economic integration, the social network in social capital can provide more employment information and economic benefits for the floating population, which in turn improves their cognitive evaluation of the quality of life (30). In terms of social adaptation, the assistance to the floating population can enhance their self-confidence and self-worth, thus promoting their social adaptation and identity, (31, 32). Social capital moderates the effect between physical health and SWB in the following way: The floating population can obtain more information about health through social networks, which is conducive to the floating population’s access to cutting-edge health information and services to ensure their physical health, enhance their emotional evaluation of their quality of life (33). Social capital moderates the effect between mental health and SWB in the following way: The trust and reciprocal norms established by social capital can help the floating population obtain more psychological counseling and psychological assistance; and this kind of emotional support helps them relieve the stress of life and maintain a good state of mind (34).

**Figure 1:**
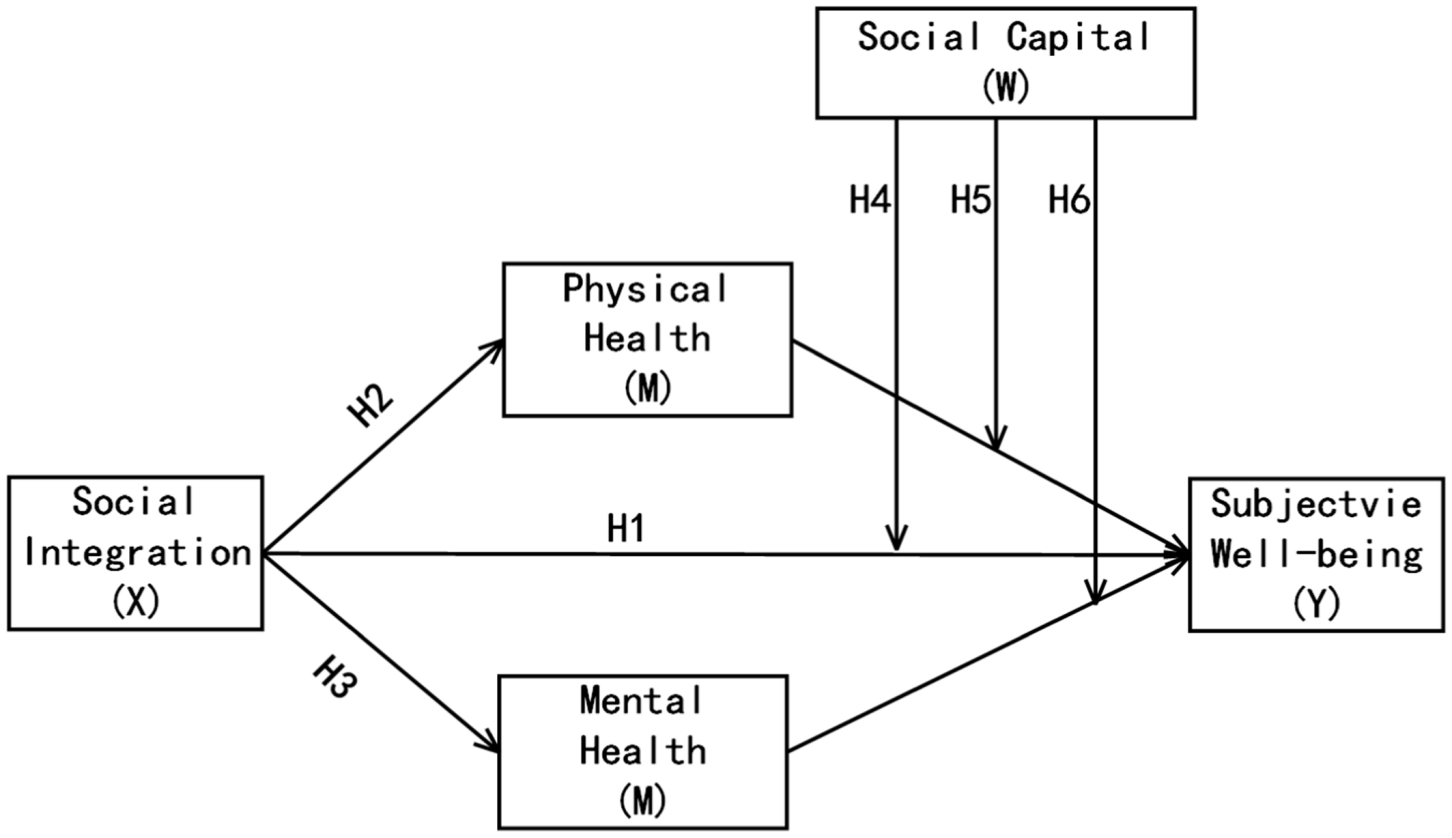
Research hypotheses and model diagram.

As shown in Figure 1, social integration is the independent variable, physical health and mental health are the mediating variables, social capital is the moderating variable, and SWB is the dependent variable. Here are my hypotheses.

*H1*: Social integration is positively correlated with SWB.

*H2*: Physical health mediates the relation between social integration and SWB.

*H3*: Mental health mediates the relation between social integration and SWB.

*H4*: Social capital can mediate the direct path of “social integration →SWB.”

*H5*: Social capital plays a positive moderating effect on the path between physical health and SWB.

*H6*: Social capital plays a positive moderating effect on the path between mental health and SWB.

## Methods

### Data collection

We use stratified, multistage method for sampling. The data collected in this study came from the Yangtze River Delta region of China in October and November 2022. As one of the regions with the highest concentration of floating population, the Yangtze River Delta region is representative, and thus was selected as the site of study. Before conducting the large-scale survey, the project team conducted a pre-survey and preliminary testing, using Cronbach’s alpha consistency coefficient to test whether the questionnaire items were consistent with the overall scale, and tested the validity through confirmatory factor analysis. We randomly choose 9 sampling sites from Zhejiang province, Jiangsu Province and Shanghai. Then, we choose three communities in each sampling site. In each selected community, we chose 20–30 people according to age, gender, and occupation. People who had migrated to Yangtze River Delta region for at least 2 years before the survey are included. The people at airports, bus and train stations are excluded by us. We trained all the investigators and provided guidance for the participant. In total, 611 valid questionnaires eventually were collected, with an effective response rate of 98%. An informed consent form was endorsed by all respondents before the survey. The measurement scale was revised according to Chinese cultural habits to ensure understanding. The investigators explained the purpose of the research to everyone. We also informed the participant that the survey was voluntary and anonymous. We give all respondents a bonus to appreciate their participation. In the study, we got the approval from Ethics Committee of Wenzhou Medical University (No. 2023–023). All data is used for research purposes only. During the research, we did not record floating population’s personal information (such as name, phone number, address), making it impossible to track individuals, and we have no possibility of causing psychological harm to them.

### Measures

#### SWB

SWB is people’s overall evaluation of their quality of life. This study used the General Well-Being Schedule (GWB) developed by the National Center for Health Statistics, which has been widely used in the measurement of SWB (35). The efficacy of the GWB is superior to other anxiety and depression scales. It includes six dimensions: well-being, self-control, vitality, depression, anxiety, and general health. Respondents were asked to rate their well-being over the past month on a 7-point scale. The scores ranged from 1 to 7, with higher scores indicating higher level of well-being.

#### Social integration

There are many ways to measure social integration. This study refers to Yue Zhongshan’s stratification basis of social integration (18), and based on the social integration measurement system proposed by Yang (36) and Zhou (37). The questionnaire uses a 5-point Likert scale that measures five dimensions of social integration.

#### Social capital

The Social Capital Scale was developed by Onyx, based on Putnam’s theory, and is divided into eight dimensions (38), including community involvement, social voluntarism, trust and security, neighborhood connection, kinship connection, diversity inclusion, life values, and work connection, for a total of 36 items. The evaluation indicators are the subjects’ feelings, reactions and levels of identification, using the 5-point Likert Scale.

#### Physical and mental health

The Health Measurement Scale, developed by the Boston Institute of Health in the United States, is a commonly used scale for measuring health status (SF-36). The current study utilized the Chinese version of the scale revised by Fang et al. (39). The scale includes two dimensions of physical and mental health, specifically including seven factors of physical function, social function, physical pain, mental health, emotional function, vitality, and general health, which can more intuitively reflect the health of participants. Respondents were asked to rated their physical and mental health over the past 4 weeks on a 6-point scale.

### Statistical strategies

In the case of controlling demographic variables, correlation analyses were conducted on independent, moderating, mediating, and dependent variables. Frequency analysis, reliability testing and Pearson correlation analysis were performed using SPSS 26.0, and validation factor models were established using Amos 22.0 to provide validity and standardized path testing. The mediating effect of physical and mental health was tested by Bootstrap.

## Results

### Descriptive statistical analysis

Table 1 illustrates the descriptive statistics. 245 (40.1%) participants were male and 366 (59.9%) were female. In terms of the distribution of education levels, 50.1% had higher education at the university level and above, 32.1% had high school education, and 17.8% reported junior high and below. Most of participants aged from 30 to 50 years old. Table 2 shows that the mean value of the question item measuring SWB was 4.27, indicating that the floating population had a medium evaluation of their own well-being. Among the influencing factors of SWB, the mean value of social integration was 3.52, indicating that the floating population had a strong willingness to integrate. The mean value of physical health was 3.55, and the mean value of mental health was 4.07, indicating that floating population’s health status was moderate to low. The mean value of social capital was 3.05, indicating that the floating population was not well connected, although they initially established some social networks in the inflow places.

**Table 1:** Descriptive statistics analysis table.

General information	*n*	Proportion (%)
**Gender**		
Male	245	40.1
Female	366	59.9
**Age**		
20–30	138	22.6
30–40	154	25.2
40–50	220	36.0
50–60	86	14.1
Over 60	13	2.1
**Education**		
Middle school and below	109	17.8
High school	196	32.1
University and above	306	50.1
**Household registration**		
Rural	351	57.4
Urban	260	42.6
**Occupation**		
Production personnel	98	16.0
Sales personnel	69	11.3
Marketing/public relations personnel	27	4.4
Customer service	24	3.9
Administrative/logistic personnel	37	6.1
HR personnel	40	6.5
Finance/audit personnel	33	5.4
Clerical/official personnel	32	5.2
Technician/developer	33	5.4
Manager	45	7.4
Teacher	47	7.7
Consultant/consulting	8	1.3
Professional skilled personnel	30	4.9
Other	88	14.4

**Table 2:** Variable mean, standard deviation, and Cronbach’s Alpha coefficient.

Variable	Mean	Standard deviation	Minimum value	Maximum value	Cronbach’s Alpha coefficient
Subjective well-being	4.2750	0.91146	1.00	7.00	0.893
Social integration	3.5244	0.69705	1.00	5.00	0.850
Social capital	3.0591	0.54871	1.00	5.00	0.894
Physical health	3.5521	1.04191	1.00	6.00	0.902
Mental health	4.0715	1.07860	1.00	6.00	0.857

### Correlation and linear regression analyses

Table 3 shows the correlation between the variables. The *p*-values corresponding to the correlation coefficients of the five latent variables involved in this paper were all less than 0.01, which is statistically significant, indicating that there were significant correlations between each of the five latent variables and they were all positively correlated. Table 4 shows the results of the regression analysis. The regression coefficients showed that none of the demographic variables in Model 1 exerted a significant effect on the SWB variable; in Model 2, the standardized regression coefficient of social integration on SWB was 0.529, *t* = 15.356, *p* < 0.001, which proved that the social integration variable had a significant positive impact on the SWB variable, so hypothesis H1 was valid.

**Table 3:** Variable correlation analysis.

	Standard deviation	Subjective well-being	Social integration	Social capital	Physical health	Mental health
Subjective well-being	0.911	1				
Social integration	0.697	0.523**	1			
Social capital	0.549	0.497**	0.511**	1		
Physical health	1.042	0.527**	0.496**	0.303**	1	
Mental health	1.079	0.595**	0.363**	0.409**	0.414**	1

**p<0.01.

**Table 4:** Multiple linear regression analysis of social integration and SWB.

	Model 1		Model 2	
	*β*	*T*	*β*	*t*
Gender	−0.031	−0.759	−0.065	−1.881
Age	0.065	1.422	0.042	1.085
Education	−0.039	−0.835	−0.07	−1.77
Household registration	0.013	0.29	0.013	0.36
Social integration			0.529	15.356***
R	0.094		0.536	
*R*^2^	0.009		0.287	
Adjusted *R*^2^	0.002		0.281	
*F*	1.345		48.654***	

***p<0.001.

### Mediating effect test

Table 5 examined the common problem of method bias in the resulting data. Using Harman’s single factor test method, the results showed that the unrotated maximum factor variance explanation rate was 39.36%, which was less than the standard of 40%, indicating that there was no single factor in the sample data that could explain most of the variance (i.e., there was no serious common method bias among the variables).

**Table 5:** Common method biases test.

Component	Initial eigenvalue			Extraction sums of squared loadings		
	Total	% of variance	Cumulative%	Total	% of variance	Cumulative%
1	9.446	39.36	39.36	9.446	39.36	39.36
2	2.616	10.901	50.261	2.616	10.901	50.261
3	1.943	8.096	58.357	1.943	8.096	58.357
4	1.256	5.234	63.592	1.256	5.234	63.592
5	1.099	4.579	68.171	1.099	4.579	68.171
24	0.194	0.809	100			

Table 6 shows the test of the mediation model. Based on Hayes’ (40) view, the bias-corrected non-parametric percentile bootstrap method was used, and the results of the mediating effect test of Model 4 in the SPSS plug-in Process showed that the total effect value of social integration on SWB was 0.684, and the 95% confidence interval was [0.595–0.773], which did not contain 0, indicating that the total effect was established; the effect value of the indirect path with physical health as a mediating variable was 0.149, and the 95% confidence interval was [0.093–0.203], which did not contain 0, indicating that the mediating effect was established and that physical health played a significant mediating role between social integration and SWB; the effect value of the indirect path with mental health as a mediating variable was 0.192, and the 95% confidence interval was [0.143–0.246], which did not contain 0, indicating that the mediating effect was established and that mental health played a significant mediating role between social integration and SWB. Therefore, support was provided for both hypotheses H2 and H3.

**Table 6:** Mediating effect test using bootstrap method.

Effect	Path	Effect size	SE	*t*	*p*	95% CI	
						LLCI	ULCI
Total effect	Social integration—subjective well-being	0.684	0.045	15.144	0.000	0.595	0.773
Direct effect	Social integration—subjective well-being	0.343	0.044	7.767	0.000	0.256	0.430
Indirect effect	Social integration—physical health—subjective well-being	0.149	0.028	–	–	0.093	0.203
	Social integration—mental health—subjective well-being	0.192	0.027	–	–	0.143	0.246

### Moderating effect test of social capital

**Figure 2:**
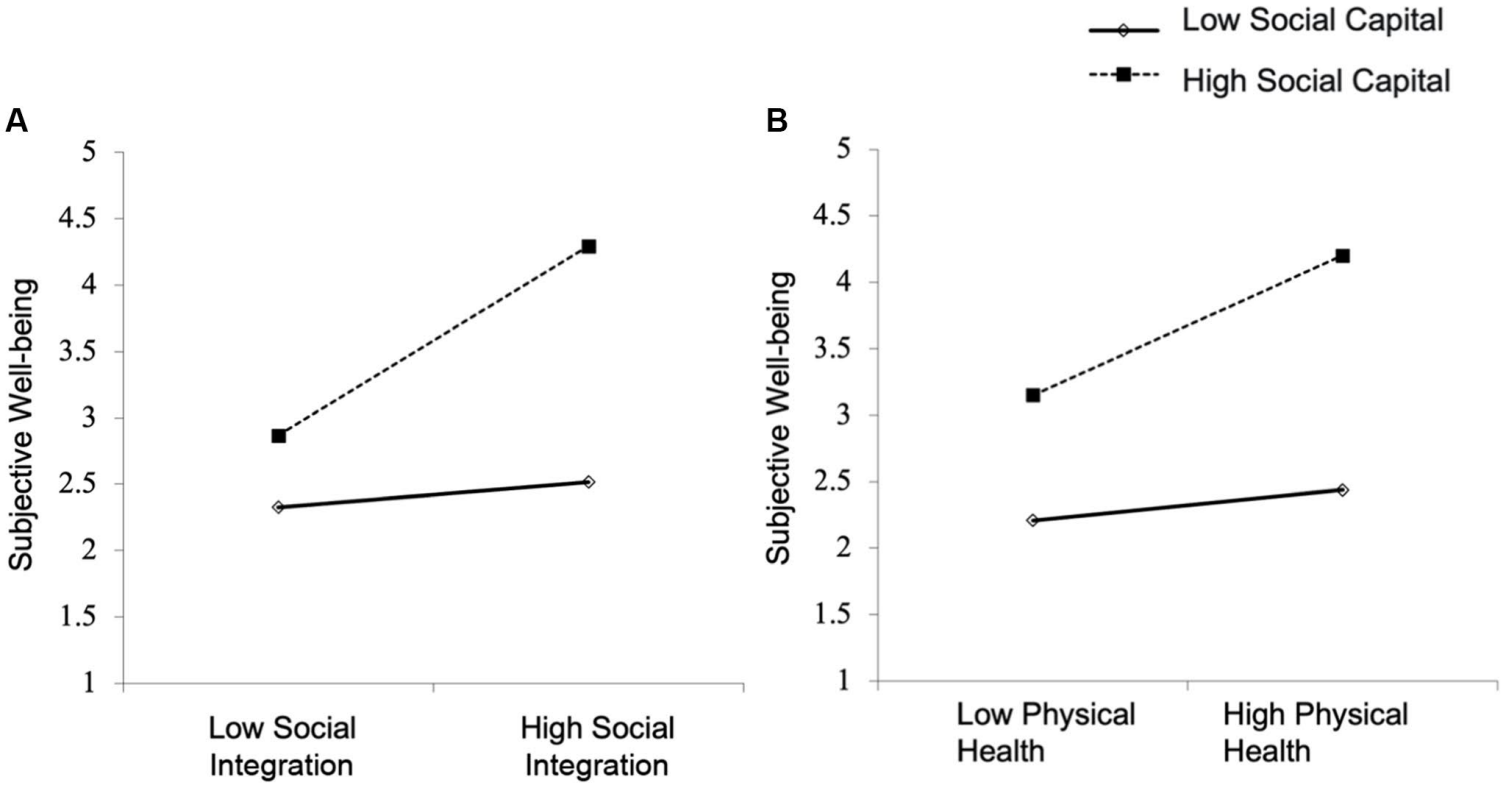
Moderating effect of social capital. **(A)** the moderating effect of social capital on the path”social integration →subjective well-being” **(B)** the moderating effect of social capital on the path “physical health → subjective well-being”.

Table 7 shows the test results of the moderating effect of social capital. This study used a hierarchical regression to test whether social capital had a moderating effect. The regression coefficient of the interaction term between the independent and moderating variables in Model 1 was 0.152 (*t* = 4.42, *p* < 0.001), indicating that the interaction term had a significant positive effect on SWB. Therefore, these results indicate that the moderating variable social capital had a significant positive moderating effect on social integration’s impact on SWB, and social capital enhanced the association between social integration and SWB. Thus, hypothesis H4 was supported. The moderating effect is shown in Figure 2A.

**Table 7:** Moderating effect test of social capital.

	Model 1 dependent variable: subjective well-being		Model 2 dependent variable: subjective well-being		Model 3 dependent variable: subjective well-being	
	*β*	*T*	*β*	*t*	*β*	*t*
Gender	−0.047	−1.438	0.008	0.247	−0.031	−1.016
Age	0.004	0.106	0.057	1.600	−0.043	−1.231
Education	−0.084	−2.269*	−0.01	−0.282	−0.066	−1.856
Household registration	−0.009	−0.244	0.009	0.268	−0.027	−0.811
Social integration	0.309	7.737***	–	–	–	–
Physical health	–	–	0.365	10.285***	–	–
Mental health	–	–	–	–	0.460	13.007***
Social capital	0.348	8.999***	0.407	11.731***	0.315	9.286***
Social integration*social capital	0.152	4.42***	–	–	–	–
Physical health*social capital	–	–	0.148	4.39**	–	–
Mental health*social capital	–	–	–	–	0.032	0.973
R	0.615		0.652		0.663	
*R*^2^	0.378		0.425		0.439	
Adjusted *R*^2^	0.371		0.419		0.433	
*F*	52.333***		63.735***		67.510***	

***p<0.001, **p<0.01, *p<0.05.

The regression coefficient of the interaction term between the independent and moderating variables in Model 2 was 0.148 (*t* = 4.39, *p* < 0.01), indicating a significant positive effect of the interaction term on SWB. Therefore, the moderating variable social capital had a significant positive moderating effect on physical health’s impact on SWB, and social capital enhanced the relationship between physical health and SWB. Thus, hypothesis H5 was supported. The moderating effect is shown in Figure 2B.

The regression coefficient of the interaction term between the independent and moderating variables in Model 3 was 0.032 (*t* = 0.973, *p* > 0.05), indicating that the interaction term did not have a significant effect on SWB. This suggests that the moderating variable of social capital did not play a significant moderating role in the effect of mental health on SWB. Therefore, hypothesis H6 was not supported.

### Moderated mediation effect model test

Table 8 shows the moderated mediation effect test with physical health as the mediating variable. Results showed that in the direct effect of social integration and SWB, when different values of social capital were taken, the 95% confidence interval of the direct effect did not contain 0, and as the level of social capital increased, the direct effect of social integration and SWB also gradually increased; in the mediation model, the confidence intervals corresponding to the indirect effects of all three levels of social capital taking values did not include 0, and the indirect role of physical health in social integration and SWB increased gradually as the level of social capital increased. This indicated that the indirect effect of physical health on social integration and SWB was moderated by different levels of social capital (i.e., a moderated mediating effect was produced).

**Table 8:** Moderated mediation effect test with physiological health as the mediating variable.

Effect	Social capital	Effect	SE	95% confidence interval	
				BootLLCI	BootULCI
Direct effect	M + 1SD	0.163	0.067	0.032	0.295
	M	0.247	0.051	0.147	0.347
	M-1SD	0.330	0.065	0.202	0.459
Indirect effect	M + 1SD	0.104	0.034	0.036	0.172
	M	0.183	0.029	0.125	0.239
	M-1SD	0.283	0.045	0.194	0.376

Table 9 shows the moderated mediation effect test with mental health as the mediating variable. The results showed that in the direct effect of social integration and SWB, when different values of social capital were taken, the 95% confidence interval of the direct effect did not contain 0, and as the level of social capital increased, the direct effect of social integration and SWB also gradually increased; in the mediation model, the confidence intervals corresponding to the indirect effects for all three levels of social capital taking values did not include 0, and as the level of social capital increased, the indirect role of mental health in social integration and SWB also gradually increased. These results indicated that the indirect effect of mental health on social integration and SWB was moderated by different levels of social capital (i.e., a moderated mediation effect was produced).

**Table 9:** Moderated mediation effect test with mental health as the mediating variable.

Effect	Social capital	Effect	SE	95% confidence interval	
				BootLLCI	BootULCI
Direct effect	M + 1SD	0.163	0.067	0.032	0.295
	M	0.247	0.051	0.147	0.347
	M-1SD	0.330	0.065	0.202	0.459
Indirect effect	M + 1SD	0.104	0.034	0.036	0.172
	M	0.183	0.029	0.125	0.239
	M-1SD	0.283	0.045	0.194	0.376

## Conclusion and discussion

In this study, we built a mediation model and explored the impact mechanism of social integration on the subjective well-being. This study can expand the research direction of immigrant health and provide reference for policy formulation and government governance. We found that five hypotheses were supported and one hypothesis was rejected. This study proved a significant correlation between social integration and SWB of Floating Population, with physical/mental health playing a mediating role. Social capital can positively moderate the association between two pair of variables: social integration and SWB. Individuals of the floating population with high willingness to socially integrate showed better SWB (11, 41), which was in line with theoretical expectations and consistent with previous research (42, 43). This is because the floating population can obtain better health resources and health care services in the process of social integration, which is conducive to promoting their physical health. Physical health is the basis of individuals’ survival in society, and the prerequisite for happiness is a pain-free and disease-free body (44). When the floating population has better physical health, their life satisfaction is higher, which in turn enhances SWB. At the same time, individuals of the floating population who have more access to more mental health education, psychological counseling and assistance, show improved emotional balance, thus enhancing their SWB.

The results of multiple hierarchical regression analysis revealed that social capital had a positive moderating effect on the path of “social integration →SWB.” Specifically, social capital can increase participation of the floating population in the social affairs of the place of migration, which can promote the SWB of the floating population (45) In addition, social capital also has a positive moderating effect on the path of “physical health → SWB.” In fact, more social capital means that the floating population can use it to obtain better community connections (27). The friendly and supportive relationship among community residents can relieve the physical burden and psychological pressure of the floating population caused by work (46). However, this study found that social capital did not have a moderating effect on the path of “mental health → SWB.” This may because the elements of mental health include social connections and positive interpersonal relationships (47), which overlap with the social network and reciprocal trust norms in social capital. In this sense, mental health reflects social capital to a certain extent. Therefore, social capital cannot moderate the relationship between mental health and SWB.

Based on the findings, this study proposes some social strategies. First, adequate employment information should be provided to the floating population to protect their labor rights and interests, thereby improving their material quality of life. Second, it is essential to cultivate their awareness and ability to participate in social affairs. Third, the government needs to monitor whether enterprises implement the health service and health care work for employees, and work to improve the community family doctor system to protect their health rights. Fourth, it is necessary to popularize health knowledge and strengthen health education, so that concepts such as regular physical examination and timely medical treatment can be better integrated into the life of the floating population. Finally, professional social organizations should strengthen external interventions, promote concepts such as neighborhood reciprocity and community mutual assistance, establish social trust norms, and increase the social capital available to floating population, thereby improving their well-being.

However, the current research does contain certain limitations. First, due to differences in well-being perception of the floating population in various regions of China, the results may be affected to some extent. Second, in terms of time, this study was a cross-sectional survey, we only investigate the floating population during a specific time period. In the future, time-series studies on the social integration and well-being can be conducted from a longitudinal perspective. Finally, this study mostly considered economic migrants from countryside to cities in China. Future research can focus on the mechanism of social integration on SWB of ecological migrants, educational immigrants, and other groups.

## Data availability statement

The raw data supporting the conclusions of this article will be made available by the corresponding author, upon reasonable request.

## Ethics statement

The studies involving human participants were reviewed and approved by The Ethics Committee of Wenzhou Medical University (NO.2022-023). The patients/participants provided their written informed consent to participate in this study.

## Author contributions

CF conducted the data analysis and drafted the preliminary manuscript. YZ participated in writing and improving the manuscript. CF, HZ, and LJ participated in measurement and data analysis. HY conceptualized and designed the study and provided suggestions for data interpretation. CF was the project coordinator and participated in all the work. All authors read and approved the final manuscript.

## Funding

This work was supported by the National Social Science Fund of China (Grant No. 21BSH036) and Key Research Center of Philosophy and Social Sciences of Zhejiang Province (Institute of Medical Humanities, Wenzhou Medical University).

## Conflict of interest

The authors declare that the research was conducted in the absence of any commercial or financial relationships that could be construed as a potential conflict of interest.

## Publisher’s note

All claims expressed in this article are solely those of the authors and do not necessarily represent those of their affiliated organizations, or those of the publisher, the editors and the reviewers. Any product that may be evaluated in this article, or claim that may be made by its manufacturer, is not guaranteed or endorsed by the publisher.
